# Innegligible musculoskeletal disorders caused by zoledronic acid in adjuvant breast cancer treatment: a meta-analysis

**DOI:** 10.1186/1756-9966-30-72

**Published:** 2011-08-04

**Authors:** Wen-Bin Zhou, Peng-Ling Zhang, Xiao-An Liu, Tao Yang, Wei He

**Affiliations:** 1 Department of Breast Surgery, The First Affiliated Hospital with Nanjing Medical University, 300 Guangzhou Road, 210029 Nanjing, China; 2 Department of Gerontology, Jiangsu Province Official Hospital, 65 Jiangsu Road, 210009 Nanjing, China; 3 Department of Endocrinology and Metabolism, The First Affiliated Hospital with Nanjing Medical University, 300 Guangzhou Road, 210029 Nanjing, China

**Keywords:** zoledronic acid, musculoskeletal disorders, breast cancer, meta-analysis

## Abstract

**Background:**

Zoledronic acid (ZOL) is widely used for preventing bone loss in early breast cancer patients. However, the adverse effects caused by ZOL itself should not be neglected. Musculoskeletal disorders were common after ZOL administration and distressing to the patients. Up to now, no precise estimation of musculoskeletal disorders has been made.

**Methods:**

Relevant randomized clinical trials were selected by searching the electronic database PubMed, and a meta-analysis was conducted.

**Results:**

Four trials reported musculoskeletal disorders of ZOL treatment versus no ZOL, including 2684 patients treated with ZOL and 2712 patients without ZOL treatment. Compared to patients without ZOL treatment, patients treated with ZOL had a significantly higher risk of arthralgia (risk ratio (RR): 1.162, 95% confidence interval (CI): 1.096-1.232, *P *= 0.466 for heterogeneity) and bone pain (RR: 1.257, 95% CI: 1.149-1.376, *P *= 0.193 for heterogeneity). Three clinical trials reported the complications of upfront versus delayed ZOL treatment, including 1091 patients with upfront ZOL and 1110 patients with delayed ZOL. The rate of bone pain in upfront group (119/824) was significantly higher than that in delayed group (74/836) (RR: 1.284, 95% CI: 1.135-1.453, *P *= 0.460 for heterogeneity).

**Conclusions:**

Our meta-analysis suggested that treatment with ZOL was significantly associated to the occurrence of arthralgia and bone pain. Moreover, higher rate of bone pain was observed in patients treated with upfront ZOL compared with delayed ZOL treatment. More attentions should be paid to patients treated with ZOL, especially for immediate ZOL. For patients with low risk of osteoporosis, immediate ZOL may be not needed due to additional musculoskeletal disorders and little benefit. Or it can be stopped after the occurrence of these adverse events.

## Introduction

More patients with early breast cancer have been diagnosed with the development of screening techniques [1]. Following adjuvant chemotherapy and endocrine therapy can significantly improve disease-free survival (DFS) and overall survival (OS) in early breast cancer patients [2-4]. However, both adjuvant chemotherapy and endocrine therapy cause bone loss to these patients. Patients with amenorrhea after chemotherapy [5,6] and postmenopausal patients receiving aromatase inhibitors (AIs) are at high risk of bone loss [3,4,7-9].

Zoledronic acid (ZOL) can prevent bone loss in early breast cancer patients [10]. Furthermore, ZOL also has antitumor and antimetastatic properties. The previous meta-analysis [11] suggested that the use of ZOL was associated with a statistically significant lower risk for disease recurrence. In addition, ZOL has several potential advantages compared to the oral bisphosphonates, including good bioavailability, gastrointestinal tolerance, and adequate compliance [12]. Thus, less adverse effects, such as gastrointestinal disorders and vascular disorders, were caused by ZOL [12]. However, the adverse effects caused by ZOL itself should not be neglected. Osteonecrosis of the jaw, an uncommon serious side effect caused by ZOL, has been paid close attention. Previous study [13] showed that osteonecrosis of the jaw occurred in only about 0.33% of patients treated with ZOL. Musculoskeletal disorders were common after ZOL administration and distressing to the patients. Up to now, no precise estimation of musculoskeletal disorders has been made. Previous randomized clinical trials [14-17] showed that musculoskeletal disorders occurred in more than 20% patients treated with ZOL and in more than 10% patients without ZOL treatment. Furthermore, some randomized trials [12,18,19] were conducted to evaluate the efficacy of upfront ZOL versus delayed ZOL in preventing bone loss. The musculoskeletal disorders reported by these trials were discordant.

The UK Expert Group [20] suggested that bisphosphonates should be administrated to patients with high risk of osteoporosis. However, patients with low risk of osteoporosis might benefit little from ZOL treatment. When ZOL was considered to be administrated to patients, the benefit and adverse effects should be well balanced. We performed this meta-analysis to give a precise estimation of the musculoskeletal disorders of ZOL versus no ZOL and upfront ZOL versus delayed ZOL in adjuvant breast cancer treatment.

## Methods

### Search strategy

The present study was conducted as described previously [21-23]. Relevant studies were selected by searching the electronic database PubMed (updated on May 1, 2011), using the following terms: early or adjuvant, breast cancer or breast neoplasm, zoledronic acid or bisphosphonates. Two investigators (Zhou WB and Liu XA) independently evaluated titles and abstracts of the identified papers. References in identified articles and reviews were also reviewed for possible inclusion. Only published randomized clinical trials in English language were included in our study. Randomized clinical trials were included if they met the following criteria: (1) ZOL used in breast cancer patients in adjuvant setting; (2) ZOL used with a control group receiving no treatment or placebo, or upfront ZOL (receiving ZOL immediately after randomization) versus delayed ZOL (receiving ZOL only if *T*-score fell below -2.0, after a nontraumatic clinical fracture, or if an asymptomatic fracture); (3) enough published data for estimated a risk ratio (RR) with 95% confidence interval (CI). In addition, to avoid duplication of information, only the report with longest follow-up was included for calculations when multiple reports pertained to overlapping groups of patients.

### Data extraction

The data of musculoskeletal disorders, including arthralgia, bone pain and muscle pain, were carefully extracted from all the eligible randomized trials independently by two investigators (Zhou WB and Liu XA). The following variables were extracted from each study: first author's name, the name of each trial, publication year, the median follow-up time, the number of total patients in every group, and the number of patients with musculoskeletal disorders in every group. All the data were reached consensus after discussion.

### Statistical analysis

Crude RRs with 95% CI were used to assess the musculoskeletal disorders risk of ZOL. The between-study heterogeneity was tested with Q statistics (significant differences indicated by *P *< 0.10) [24]. The fixed-effects model (the Mantel-Haenszel method) was used when between-study was absent [25]. Otherwise, the random-effects model (the DerSimonian and Laird method) was selected [26]. Funnel plots and Egger's linear regression were used to test the publication bias and a *P *value less than 0.05 was considered significant. All analyses were performed using the software Stata version 11.0 (Stata Corporation, College Station, TX, USA).

## Results

### Eligible studies

Ten randomized clinical trials, in which ZOL was used in adjuvant setting, were identified. Of these ten studies, the detail data of musculoskeletal disorders were not reported in three studies [27-29]. In all, seven studies [12,14-19] were eligible in this meta-analysis. Table 1 presented the characteristics of the seven trials. Of these seven studies, four studies [14-17] reported musculoskeletal disorders of ZOL versus placebo or no treatment, including 2684 patients treated with ZOL and 2712 patients treated with placebo or no treatment. Three studies [12,18,19] reported the complications of upfront versus delayed ZOL, including 1091 patients with upfront ZOL and 1110 patients with delayed ZOL.

**Table 1:** Characteristics of eligible trials

Author (Study)	Year	Intervention	Dosage of treatment	Duration (yr)	Number of patients	Follow-up (mo)
Gnant (ABCSG12)	2009	Zoledronic acid No treatment	4 mg IV every 6 months	3	899 904	47.8
Shapiro (CALGB)	2011	Zoledronic acid No treatment	4 mg IV every 3 months	NA	70 80	12
Hershman	2008	Zoledronic acid Placebo	4 mg IV every 3 months	1	50 53	12
Coleman (AZURE)	2011	Zoledronic acid No treatment	4 mg IV monthly for 6 months, then every 3 months for 8 doses and then every 6 months for 5 doses	5	1665 1675	6
Brufsky (Z-FAST)	2009	Upfront zoledronic acid Delayed zoledronci acid	4 mg IV every 6 months	5	300 300	36
Eidtmann (ZO-FAST)	2010	Upfront zoledronic acid Delayed zoledronci acid	4 mg IV every 6 months	5	524 536	36
Hines (N03CC)	2009	Upfront zoledronic acid Delayed zoledronci acid	4 mg IV every 6 months	5	267 274	12

yr, year; mo, months; IV, intravenous; NA, not available

### ZOL versus no ZOL

**Figure 1:**
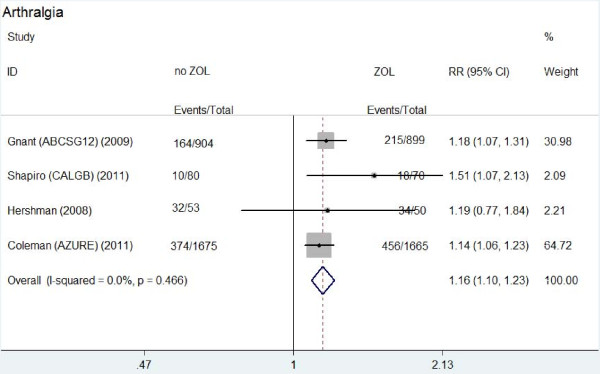
**Forest plot for meta-analysis of arthralgia of patients treated with zoledronic acid (ZOL) versus no ZOL**.

**Figure 2:**
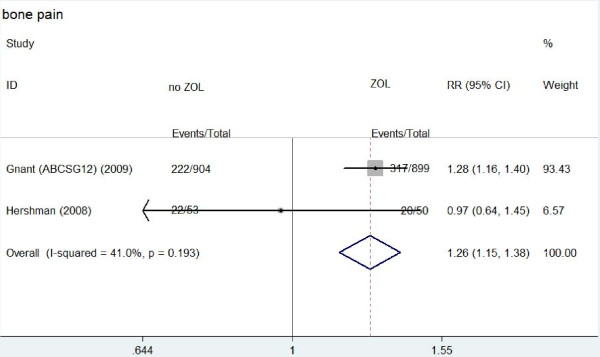
**Forest plot for meta-analysis of bone pain of patients treated with zoledronic acid (ZOL) versus no ZOL**.

Table 2 showed the main results of this meta-analysis. Arthralgia occurred in about 23.9%-68% patients treated with ZOL and 12.5%-60.4% patients without ZOL treatment. Compared to patients without ZOL treatment, patients treated with ZOL had a significantly higher risk of arthralgia (RR: 1.162, 95% CI: 1.096-1.232, *P *= 0.466 for heterogeneity) (Figure 1). Bone pain occurred in about 35.3%-40% patients treated with ZOL and in 24.6%-41.5% patients without ZOL treatment. Similarly, a significantly higher risk of bone pain was observed in patients with ZOL treatment (RR: 1.257, 95% CI: 1.149-1.376, *P *= 0.193 for heterogeneity) (Figure 2). However, there was no significantly different risk of muscle pain between the two groups (RR: 1.198, 95% CI: 0.901-1.594, *P *= 0.366 for heterogeneity).

**Table 2:** Summary RRs and 95% CI

Complications	ZOL vs no ZOL			Upfront ZOL vs delayed ZOL		
	RR (95%CI)	*P*^⋆^	Number of studies	RR (95%CI)	*P*^⋆^	Number of studies
Arthralgia	**1.162 (1.096-1.232)^#^**	0.466	4	1.022 (0.932-1.120)	0.850	3
Bone pain	**1.257 (1.149-1.376)**	0.193	2	**1.284 (1.135-1.453)**	0.460	2
Muscle pain	1.198 (0.901-1.594)	0.366	2	1.071 (0.942-1.217)	0.422	3

RR, risk ratio; CI, confidence interval; ZOL, zoledronic acid;

*P value for between-study heterogeneity; #the number in AZURE trial included the number of arthralgia and muscle pain.

Funnel plot and Egger's test were performed to access the publication bias of the four studies. No significant publication bias (*P *> 0.05) existed (data not shown).

### Upfront versus delayed-start ZOL

**Figure 3:**
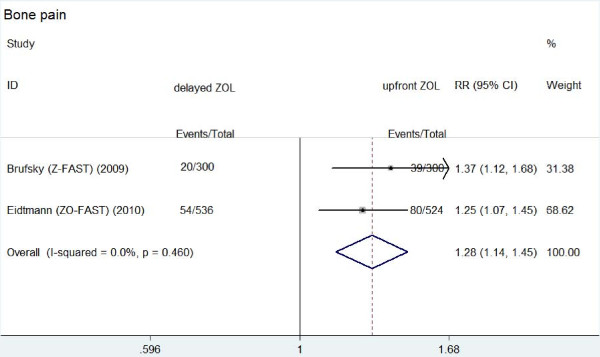
**Forest plot for meta-analysis of bone pain of patients treated with upfront zoledronic acid (ZOL) versus delayed ZOL**.

The main results were also showed in Table 2. Arthralgia occurred in 12.7%-42.2% patients treated with upfront ZOL and in 11.3%-40.7% patients with delayed ZOL. There was no significantly different risk of arthralgia between the two groups (RR: 1.022, 95% CI: 0.932-1.120, *P *= 0.850 for heterogeneity). The similar results were observed about muscle pain between the two groups (RR: 1.071, 95% CI: 0.942-1.217, *P *= 0.422 for heterogeneity). The rates of muscle pain were 6.4%-16.3% and 5.1%-12.1% in upfront group and delayed group, respectively. Bone pain caused by ZOL was reported in Z-FAST and ZO-FAST trials. The rate of bone pain in upfront group (119/824) was significantly higher than that in delayed group (74/836) (RR: 1.284, 95% CI: 1.135-1.453, *P *= 0.460 for heterogeneity) (Figure 3).

Since only three trials were included in this analysis of musculoskeletal disorders between upfront and delayed ZOL groups, publication bias was not accessed.

## Discussion

Previous randomized clinical trials showed that musculoskeletal disorders occurred in a high rate of patients treated with ZOL. This meta-analysis suggested that patients treated with ZOL had a statistically significant higher risk of arthralgia and bone pain compared to patients without ZOL treatment. Furthermore, patients treated with upfront ZOL had a significant higher risk of bone pain than patients with delayed ZOL.

Although ZOL can bypass the potential disadvantages of the oral route used by other bisphosphonates, it may cause more musculoskeletal disorders than other bisphosphonates [30-32]. A high rate of musculoskeletal disorders occurred in patients treated with ZOL. Patients treated with ZOL had a statistically significant higher risk of arthralgia and bone pain than patients without ZOL treatment. These adverse effects bring anxiety to patients and may threaten patients' life quality in some conditions. These adverse effects generally resolve within 48 hours and respond well to nonsteroidal anti-inflammatory drugs [33]. Of these patients, some suffered serious musculoskeletal disorders from ZOL treatment, which exist longer and respond worse to anti-inflammatory drugs. Sometimes, serious musculoskeletal disorders cause treatment withdrawal. Although most musculoskeletal disorders will disappear spontaneously, we should take more attentions to patients treated with ZOL. The dose, frequency, and speed of infusion are all important determinants of these adverse effects [33]. When patients with high risk of osteoporosis suffered serious musculoskeletal disorders from ZOL, the risk-reducing measures should be considered. These measures included reducing the dose, slowing the infusion rate and prolonging the interval between infusions. When the patients can not tolerate these adverse effects, other oral bisphosphonates should be considered [33]. When ZOL was administrated to patients with low risk of osteoporosis, little benefit but additional musculoskeletal disorders would be brought to these patients.

Three randomized clinical trials [12,18,19] were conducted to compare upfront ZOL with delayed ZOL for prevention of bone loss in postmenopausal women. These studies suggested that upfront ZOL was more effective in preserving bone mineral density than delayed ZOL, but no significant difference in fracture rate was observed. The UK Expert Group [20] suggested that patients with low risk of osteoporosis did not need a special treatment, while patients with high risk should be treated with bisphosphonates. Our results suggested more musculoskeletal disorders were observed in patients treated with upfront ZOL. Since not all patients need upfront ZOL treatment, delayed ZOL may be considered preferentially in some conditions. In addition, although ZO-FAST trial showed that upfront ZOL led to improved DFS, further randomized trials are required to investigate the survival and adverse effects between upfront ZOL and delayed ZOL.

Several limitations of this meta-analysis should be considered when interpreting these results. First, of these seven studies, most subjects were Caucasians, while seldom Asians were included. Second, the present results were based on unadjusted RRs. More precise estimation may be adjusted by other potential covariates. Third, due to lack of data on musculoskeletal disorders, three trials were excluded. Since these studies were with small sample size, they were unlikely to change significantly our results.

## Conclusions

This meta-analysis strongly suggested that patients treated with ZOL had a statistically significant higher risk of arthralgia and bone pain than those without ZOL treatment. Furthermore, patients treated with upfront ZOL had a significantly higher risk of bone pain than patients with delayed ZOL. More attentions should be paid to patients with musculoskeletal disorders. For patients with low risk of osteoporosis, immediate ZOL may be not needed due to additional adverse effects in some conditions. Or it can be stopped after the occurrence of these adverse events. Further randomized clinical trials with large sample size should be taken to evaluate the side effects of ZOL, especially for musculoskeletal disorders.

## List of abbreviations

AI: aromatase inhibitor; CI: confidence interval; DFS: disease-free survival; OS: overall survival; RR: risk ratio; ZOL: zoledronic acid.

## Conflict of interest

The authors declare that they have no competing interests.

## Authors' contributions

WH has contributed to the conception and design of the study, the analysis and interpretation of data, the revision of the article as well as final approval of the version to be submitted. WBZ and XAL participated in the design of the study, performed the statistical analysis, searched and selected the trials, drafted and revised the article. PLZ drafted and revised the article. TY participated in the design of the study and helped to revise the article. All authors read and approved the final version of the manuscript.

## References

[B1] Elmore JG Armstrong K Lehman CD Fletcher SW Screening for breast cancer JAMA 2005 293 1245 1256 10.1001/jama.293.10.1245 15755947 PMC3149836

[B2] Early Breast Cancer Trialists' Collaborative Group (EBCTCG) Effects of chemotherapy and hormonal therapy for early breast cancer on recurrence and 15-year survival: an overview of the randomised trials Lancet 2005 365 1687 1717 15894097 10.1016/S0140-6736(05)66544-0

[B3] Forbes JF Cuzick J Buzdar A Howell A Tobias JS Baum M Effect of anastrozole and tamoxifen as adjuvant treatment for early-stage breast cancer: 100-month analysis of the ATAC trial Lancet Oncol 2008 9 45 53 18083636 10.1016/S1470-2045(07)70385-6

[B4] Coates AS Keshaviah A Thurlimann B Mouridsen H Mauriac L Forbes JF Paridaens R Castiglione-Gertsch M Gelber RD Colleoni M Lang I Del Mastro L Smith I Chirgwin J Nogaret JM Pienkowski T Wardley A Jakobsen EH Price KN Goldhirsch A Five years of letrozole compared with tamoxifen as initial adjuvant therapy for postmenopausal women with endocrine-responsive early breast cancer: update of study BIG 1-98 J Clin Oncol 2007 25 486 492 10.1200/JCO.2006.08.8617 17200148

[B5] Di Cosimo S Alimonti A Ferretti G Sperduti I Carlini P Papaldo P Fabi A Gelibter A Ciccarese M Giannarelli D Mandalà M Milella M Ruggeri EM Cognetti F Incidence of chemotherapy-induced amenorrhea depending on the timing of treatment by menstrual cycle phase in women with early breast cancer Ann Oncol 2004 15 1065 1071 10.1093/annonc/mdh266 15205200

[B6] Zhou WB Yin H Liu XA Zha XM Chen L Dai JC Tao AD Ma JJ Ling LJ Wang S Incidence of chemotherapy-induced amenorrhea associated with epirubicin, docetaxel and navelbine in younger breast cancer patients BMC Cancer 2010 10 281 10.1186/1471-2407-10-281 20540745 PMC2893114

[B7] Fuleihan Gel H Salamoun M Mourad YA Chehal A Salem Z Mahfoud Z Shamseddine A Pamidronate in the prevention of chemotherapy-induced bone loss in premenopausal women with breast cancer: a randomized controlled trial J Clin Endocrinol Metab 2005 90 3209 3214 10.1210/jc.2004-1444 15769994

[B8] Shapiro CL Manola J Leboff M Ovarian failure after adjuvant chemotherapy is associated with rapid bone loss in women with early-stage breast cancer J Clin Oncol 2001 19 3306 3311 11454877 10.1200/JCO.2001.19.14.3306

[B9] Simpson ER Dowsett M Aromatase and its inhibitors: significance for breast cancer therapy Recent Prog Horm Res 2002 57 317 338 10.1210/rp.57.1.317 12017550

[B10] Jansen JP Bergman GJ Huels J Olson M The efficacy of bisphosphonates in the prevention of vertebral, hip, and nonvertebral-nonhip fractures in osteoporosis: a network meta-analysis Semin Arthritis Rheum 2011 40 275 284 e271-272 10.1016/j.semarthrit.2010.06.001 20828791

[B11] Mauri D Valachis A Polyzos NP Tsali L Mavroudis D Georgoulias V Casazza G Does adjuvant bisphosphonate in early breast cancer modify the natural course of the disease? A meta-analysis of randomized controlled trials J Natl Compr Canc Netw 2010 8 279 286 20202461 10.6004/jnccn.2010.0020

[B12] Hines SL Mincey B Dentchev T Sloan JA Perez EA Johnson DB Schaefer PL Alberts S Liu H Kahanic S Mazurczak MA Nikcevich DA Loprinzi CL Immediate versus delayed zoledronic acid for prevention of bone loss in postmenopausal women with breast cancer starting letrozole after tamoxifen-N03CC Breast Cancer Res Treat 2009 117 603 609 10.1007/s10549-009-0332-2 19214743 PMC3907065

[B13] Mauri D Valachis A Polyzos IP Polyzos NP Kamposioras K Pesce LL Osteonecrosis of the jaw and use of bisphosphonates in adjuvant breast cancer treatment: a meta-analysis Breast Cancer Res Treat 2009 116 433 439 10.1007/s10549-009-0432-z 19521766

[B14] Gnant M Mlineritsch B Schippinger W Luschin-Ebengreuth G Pöstlberger S Menzel C Jakesz R Seifert M Hubalek M Bjelic-Radisic V Samonigg H Tausch C Eidtmann H Steger G Kwasny W Dubsky P Fridrik M Fitzal F Stierer M Rücklinger E Greil R ABCSG-12 Trial Investigators Marth C Endocrine therapy plus zoledronic acid in premenopausal breast cancer N Engl J Med 2009 360 679 691 10.1056/NEJMoa0806285 19213681

[B15] Shapiro CL Halabi S Hars V Archer L Weckstein D Kirshner J Sikov W Winer E Burstein HJ Hudis C Isaacs C Schilsky R Paskett E Zoledronic acid preserves bone mineral density in premenopausal women who develop ovarian failure due to adjuvant chemotherapy: final results from CALGB trial 79809 Eur J Cancer 2011 47 683 689 10.1016/j.ejca.2010.11.024 21324674 PMC4211594

[B16] Hershman DL McMahon DJ Crew KD Cremers S Irani D Cucchiara G Brafman L Shane E Zoledronic acid prevents bone loss in premenopausal women undergoing adjuvant chemotherapy for early-stage breast cancer J Clin Oncol 2008 26 4739 4745 10.1200/JCO.2008.16.4707 18711172 PMC2653138

[B17] Coleman R Woodward E Brown J Cameron D Bell R Dodwell D Keane M Gil M Davies C Burkinshaw R Houston SJ Grieve RJ Barrett-Lee PJ Thorpe H Safety of zoledronic acid and incidence of osteonecrosis of the jaw (ONJ) during adjuvant therapy in a randomised phase III trial (AZURE: BIG 01-04) for women with stage II/III breast cancer Breast Cancer Res Treat 2011 127 429 438 10.1007/s10549-011-1429-y 21394500

[B18] Brufsky AM Bosserman LD Caradonna RR Haley BB Jones CM Moore HC Jin L Warsi GM Ericson SG Perez EA Zoledronic acid effectively prevents aromatase inhibitor-associated bone loss in postmenopausal women with early breast cancer receiving adjuvant letrozole: Z-FAST study 36-month follow-up results Clin Breast Cancer 2009 9 77 85 10.3816/CBC.2009.n.015 19433387

[B19] Eidtmann H de Boer R Bundred N Llombart-Cussac A Davidson N Neven P von Minckwitz G Miller J Schenk N Coleman R Efficacy of zoledronic acid in postmenopausal women with early breast cancer receiving adjuvant letrozole: 36-month results of the ZO-FAST Study Ann Oncol 2010 21 2188 2194 10.1093/annonc/mdq217 20444845

[B20] Reid DM Prevention of osteoporosis after breast cancer Maturitas 2009 64 4 8 10.1016/j.maturitas.2009.07.008 19709826

[B21] Zhou WB Xue DQ Liu XA Ding Q Wang S The influence of family history and histological stratification on breast cancer risk in women with benign breast disease: a meta-analysis J Cancer Res Clin Oncol 2011 137 1053 1060 10.1007/s00432-011-0979-z 21499874 PMC3112325

[B22] Zhou WB Ding Q Chen L Liu XA Wang S Toremifene is an effective and safe alternative to tamoxifen in adjuvant endocrine therapy for breast cancer: results of four randomized trials Breast Cancer Res Treat 2011 128 625 631 10.1007/s10549-011-1556-5 21553116

[B23] Liu X Wang Z Yu J Lei G Wang S Three polymorphisms in interleukin-1beta gene and risk for breast cancer: a meta-analysis Breast Cancer Res Treat 2010 124 821 825 10.1007/s10549-010-0910-3 20437198

[B24] Lau J Ioannidis JP Schmid CH Quantitative synthesis in systematic reviews Ann Intern Med 1997 127 820 826 9382404 10.7326/0003-4819-127-9-199711010-00008

[B25] Mantel N Haenszel W Statistical aspects of the analysis of data from retrospective studies of disease J Natl Cancer Inst 1959 22 719 748 13655060

[B26] DerSimonian R Laird N Meta-analysis in clinical trials Control Clin Trials 1986 7 177 188 10.1016/0197-2456(86)90046-2 3802833

[B27] Leal T Tevaarwerk A Love R Stewart J Binkley N Eickhoff J Parrot B Mulkerin D Randomized trial of adjuvant zoledronic acid in postmenopausal women with high-risk breast cancer Clin Breast Cancer 2010 10 471 476 10.3816/CBC.2010.n.062 21147691 PMC3091169

[B28] Swenson KK Nissen MJ Anderson E Shapiro A Schousboe J Leach J Effects of exercise vs bisphosphonates on bone mineral density in breast cancer patients receiving chemotherapy J Support Oncol 2009 7 101 107 19507458

[B29] Kim JE Ahn JH Jung KH Kim SB Kim HJ Lee KS Ro JS Park YH Ahn JS Im YH Im SA Lee MH Kim SY Zoledronic acid prevents bone loss in premenopausal women with early breast cancer undergoing adjuvant chemotherapy: a phase III trial of the Korean Cancer Study Group (KCSG-BR06-01) Breast Cancer Res Treat 2011 125 99 106 10.1007/s10549-010-1201-8 20922564

[B30] Van Poznak C Hannon RA Mackey JR Campone M Apffelstaedt JP Clack G Barlow D Makris A Eastell R Prevention of aromatase inhibitor-induced bone loss using risedronate: the SABRE trial J Clin Oncol 2010 28 967 975 10.1200/JCO.2009.24.5902 20065185

[B31] Hines SL Mincey BA Sloan JA Thomas SP Chottiner E Loprinzi CL Carlson MD Atherton PJ Salim M Perez EA Phase III randomized, placebo-controlled, double-blind trial of risedronate for the prevention of bone loss in premenopausal women undergoing chemotherapy for primary breast cancer J Clin Oncol 2009 27 1047 1053 10.1200/JCO.2008.19.1783 19075260 PMC2667810

[B32] Markopoulos C Tzoracoleftherakis E Polychronis A Venizelos B Dafni U Xepapadakis G Papadiamantis J Zobolas V Misitzis J Kalogerakos K Sarantopoulou A Siasos N Koukouras D Antonopoulou Z Lazarou S Gogas H Management of anastrozole-induced bone loss in breast cancer patients with oral risedronate: results from the ARBI prospective clinical trial Breast Cancer Res 2010 12 R24 10.1186/bcr2565 20398352 PMC2879572

[B33] Diel IJ Bergner R Grotz KA Adverse effects of bisphosphonates: current issues J Support Oncol 2007 5 475 482 18240669

